# Antimicrobial Effect of Honey Phenolic Compounds against *E. coli*—An In Vitro Study

**DOI:** 10.3390/ph17050560

**Published:** 2024-04-27

**Authors:** Laura Kassym, Assiya Kussainova, Yuliya Semenova, Pauline McLoone

**Affiliations:** 1 Department of General Medical Practice with a Course of Evidence-Based Medicine, NJSC “Astana Medical University”, Astana 010000, Kazakhstan; laura.kassym@gmail.com; 2 School of Medicine, Nazarbayev University, Astana 010000, Kazakhstan; a.kussainova@nu.edu.kz; 3 School of Medicine, University of Kurdistan Hewler, Erbil 44001, Iraq; pauline.mcloone@ukh.edu.krd

**Keywords:** honey, antibacterial, 3-phenyllactic acid, *p*-coumaric acid, phloretin, *E. coli* (ATCC25922)

## Abstract

Growing concern over antimicrobial resistance in chronic wound patients necessitates the exploration of alternative treatments from natural sources. This study suggests that honey’s phenolic compounds may offer antimicrobial benefits, warranting further investigation for therapeutic development. The main aim of this study was to investigate the antimicrobial activity of phenolic compounds and to determine the effects of their sub-inhibitory concentrations against *Escherichia coli* (*E. coli*). 3-phenyllactic acid (PLA), *p*-coumaric acid (PCA), and phloretin were tested against the bacterial strain of *E. coli* ATCC 25922. Comparison of the antimicrobial activity of honey constituents in vitro was performed using a broth culture assay. Measurement of the inhibitory properties of constituents in vitro was conducted using disc and well diffusion assays. The effects of sub-inhibitory concentrations of PCA on the susceptibility of *E. coli* ATCC 25922 to penicillin–streptomycin were tested. The results demonstrated that PLA was the most efficient antimicrobial agent, followed by PCA, whereas phloretin, at lower (2 mg/mL) concentrations, led to an increase in the growth of *E. coli*. Various modifications of the agar diffusion assay did not reveal the antibacterial properties of the studied phytochemicals. The enhancing effect of a sub-inhibitory concentration of PCA in cooperation with penicillin–streptomycin was shown. These findings might be helpful for the further investigation and development of new antimicrobial agents for the treatment of skin infections and wounds.

## 1. Introduction

The extensive historical use of honey spans both medical and cosmetic applications [1]. It has been utilized to address a range of health issues including eye ailments, asthma, throat infections, tuberculosis, and skin conditions like eczema [2,3]. Additionally, honey serves as a nutritional supplement [4]. While its primary use is in skin diseases and wound healing due to its perceived efficacy, honey’s benefits extend to reducing edema, inflammation, and pain, fostering regeneration, and providing wound deodorization [5,6]. Research by Al-Waili (2001, 2003, 2004) on UAE honey demonstrates its effectiveness in treating dermatological conditions such as atopic dermatitis, psoriasis, fungal infections, and herpes simplex [7,8,9]. 

Honey is a complex solution with primary constituents including high concentrations of sugars like fructose (around 38.2%), glucose (approximately 31.3%), and sucrose (1%), along with significant water content (about 17%) [10]. While these main components remain consistent across different types of honey, minor constituents vary greatly depending on geographical origin and floral source [11]. These minor compounds include proteins, amino acids (up to 0.5%), organic acids, vitamins (such as A, B2, B6, and C), enzymes (like diastase, invertase, glucose oxidase, and catalase), carotenoid derivatives, minerals, flavonoids, and polyphenols [12]. Several studies using standardized honeys have determined minimum inhibitory concentration (MIC) values for various microbes commonly found in infected wounds, such as *Staphylococcus aureus* (*S. aureus*), different coagulase-negative *Staphylococci*, various *Streptococci species*, different *Enterococci species*, *Pseudomonas aeruginosa* (*P. aeruginosa*), *Escherichia coli* (*E. coli*), *Klebsiella oxytoca* (*K. oxytoca*), and anaerobes [13,14,15]. Honey’s antimicrobial properties result from its high osmolarity, inhibiting bacterial growth, low acidity (pH 3.4–6.1), and its production of hydrogen peroxide during glucose oxidation by honeybees [16]. However, in some cases, honey’s antibacterial effectiveness is not solely dependent on sugar concentration or hydrogen peroxide production [17]. Manuka honey’s high levels of phenolic acids, flavonoids, and other compounds, e.g., methylglyoxal (MGO), greatly boost its antibacterial effectiveness [18,19]. Recent studies have highlighted the broad-spectrum antimicrobial properties of 3-phenyllactic acid (PLA), found in Manuka honey [20,21,22]. In a study conducted by Ning et al. (2017), the authors illustrated that the targets of PLA against foodborne pathogenic bacteria were the bacterial membrane and genomic DNA. However, the authors observed that in the case of *E. coli*, PLA could only penetrate the outer membrane of the bacterium without compromising the permeability of its inner wall [23]. The next relevant component of Manuka honey is *p*-coumaric acid (PCA), which also exhibits antimicrobial properties through the disruption of bacterial cell walls and interference with microbial DNA [24,25,26,27]. Phloretin, another component of Manuka honey, possesses both antimicrobial and anti-inflammatory effects [28]. Phloretin showed bacteriostatic and antibiofilm effects against various bacteria, including *Haemophilus influenzae*, *Moraxella catarrhalis*, *Streptococcus pneumoniae*, and *P. aeruginosa* [29]. Another study showed its superior antibacterial activity against *Cutibacterium acnes* compared to benzoyl peroxide, a standard acne treatment [30]. 

The rising interest in the antibacterial attributes of honey stems primarily from the escalating challenge of antimicrobial resistance, prompting the quest for novel alternative topical therapies for wound infections [10,31,32,33]. Infections caused by antibiotic-resistant strains directly correlate with diminished quality of life, heightened rates of recurrent infections, elevated instances of treatment failure, and increased susceptibility to complications [34]. Among Gram-negative bacterial pathogens, *E. coli* stands out as the most prevalent, posing a substantial burden on both clinical medicine and public health [35]. *E. coli* strains have demonstrated resistance to specific antimicrobial groups such as third-generation cephalosporins, fluoroquinolones, and aminoglycosides [34]. Additionally, Basualdo et al. (2007) observed that *E. coli* exhibited heightened resistance to various honey samples compared to Gram-positive bacteria [36]. Therefore, investigating the antibacterial attributes of honey’s chemical constituents may offer potential solutions to the challenge of antimicrobial resistance.

Thus, the primary aim of this investigation was to examine the antimicrobial effects of PLA, PCA, and phloretin against *E. coli.* using a broth culture assay and disk and well diffusion assays. The secondary aim was to examine the effect of sub-inhibitory concentrations of PCA on the susceptibility of *E. coli* to penicillin/streptomycin. 

## 2. Results

### 2.1. Comparison of the Antimicrobial Activity of Honey Phytochemicals In Vitro Using a Broth Culture Assay

**Figure 1:**
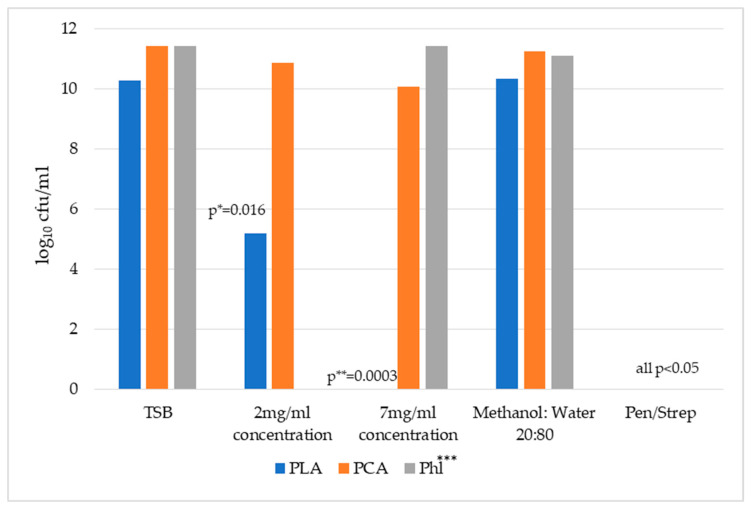
Effect of honey phenolic compounds on the growth of *E. coli* (ATCC 25922). Results are expressed as the mean SEM of log_10_ cfu/mL. Experimental data for each concentration of phenolic compounds, methanol/sterile water solvent (20%/80%), and penicillin–streptomycin (2000U penicillin; 2 mg/mL streptomycin) solvent were compared to the TSB growth control. * Results are significantly different from TSB control; ** sesult for PLA at 7 mg/mL concentration; *** phloretin at 2 mg/mL concentration was associated with the exceed growth of *E. coli* in all rounds of the experiment. TSB—tryptone soy broth; PCA—3-phenyllactic acid; PCA—*p*-coumaric acid; Phl—phloretin.

Figure 1 demonstrates the results of *E. coli* growth in broths of all tested phenolic compounds at two investigated concentrations and controls. PLA at 7 mg/mL and 2 mg/mL concentrations significantly inhibited the growth of *E. coli.* Mean log_10_ cfu/mL were 0 and 5.16, respectively (all *p* < 0.05). The difference between PCA at 2 mg/mL concentration and TSB control was not statistically significant (*p* = 0.1092). The PCA at 7 mg/mL concentration demonstrated significantly less bacteria; growth than the TSB alone; the mean log_10_ cfu/mL was 10.06 and 11.41, respectively (*p* = 0.0091). The phloretin at 7 mg/mL concentration did not significantly decrease the growth of *E. coli*. Throughout all stages of the experiment, *E. coli* exhibited increased growth when subjected to a concentration of 2 mg/mL of phloretin. Furthermore, the comparison between concentrations of 2 mg/mL and 7 mg/mL for both PLA and PCA does not yield statistically significant findings.

### 2.2. Screening for the Antimicrobial Activity of Honey Constituents—Disk Diffusion Assay

Table 1 demonstrates the antibacterial properties of honey constituents at 100 μg per disc. All phytochemicals had no antimicrobial power against *E. coli*, whereas both positive controls had visible zones of inhibition. 

Then, the disc diffusion assay for the investigation of the antibacterial capacity of phytochemicals at 350 μg per disc was performed. As is shown in Table 2, even at increased concentrations, none of the tested phytochemicals inhibited the growth of *E. coli.* Again, penicillin–streptomycin and methylglyoxal demonstrated antibacterial effects.

### 2.3. Assessment of the Antimicrobial Activity of Honey Constituents—Well Diffusion Assay

Table 3 shows that the investigated phytochemicals at 700 μg per well had no activity against *E. coli*. The average diameter of zones of inhibition from four replicates of penicillin–streptomycin was 33.50 ± 2.20 mm. Methylglyoxal had a less-inhibitory effect against *E. coli* (31.78 ± 1.70 mm), but the difference was not significant (*p* = 0.055).

### 2.4. Determination of the Effects of Sub-Inhibitory Concentrations of p-Coumaric Acid on the Susceptibility of E. coli to Penicillin–Streptomycin

**Figure 2:**
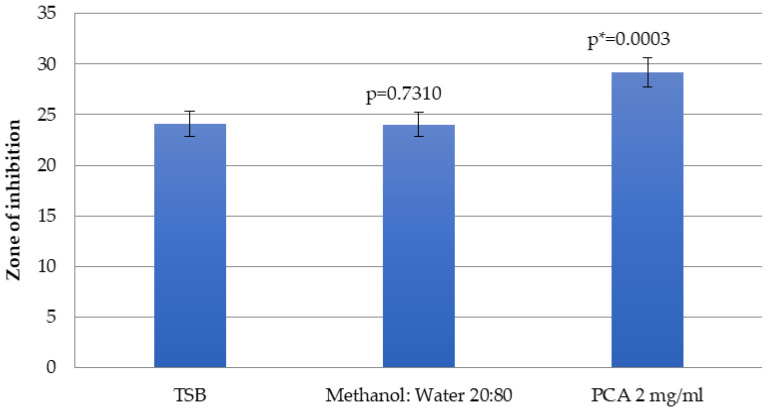
Effect of penicillin–streptomycin solvent on the growth of *E. coli* (ATCC 25922) in combination with *p*-coumaric acid. Results are expressed as the mean with the SEM of zone of inhibition after incubation at 37 °C for 24 h. Experimental data for *p*-coumaric acid at 2 mg/mL concentration and methanol/sterile water solvent (20%/80%) were compared to the TSB growth control. * Results are significantly different from TSB control.

Figure 2 shows the inhibitory effects of penicillin–streptomycin in cooperation with *p*-coumaric acid at 2 mg/mL concentration. This phytochemical demonstrates the significant decrease in growth of *E. coli*, supporting the inhibitory action of the antibiotics. The mean zone of inhibition induced by the penicillin–streptomycin solvent when the bacteria were previously incubated with 2 mg/mL of *p*-coumaric acid was 29.2 ± 1.3 mm, whereas the zone of inhibition for the TSB control was 24.1 ± 1.7 mm (*p* = 0.0003).

## 3. Discussion

In this study, the antibacterial properties of honey phytochemicals against *E. coli* (ATCC 25922) were investigated. The data obtained from the broth culture assay demonstrated that several honey constituents significantly decreased the growth of *E. coli* after incubation at 37 °C for 24 h. It appears that *E. coli* was more sensitive to the antimicrobial effects of PLA than to the antimicrobial action of PCA and phloretin. PLA at 7 mg/mL and 2 mg/mL concentrations significantly inhibited the growth of *E. coli* (mean log10 cfu/mL was 0 and 5.16, respectively (all *p* < 0.05)). PCA at different concentrations demonstrated various effects on the growth of *E. coli*. In this case, at a 2 mg/mL concentration, PCA did not exhibit significant inhibitory activity (*p* = 0.1092 in comparison with the mean log10 cfu/mL of TSB broth control). However, at a 7 mg/mL concentration, PCA demonstrated significantly less growth of *E. coli* than the TSB alone; the mean log10 cfu/mL was 10.06 and 11.41, respectively (*p* = 0.0091). 

There are several studies dedicated to testing the antimicrobial potency of the different honeys against *E. coli* using some modifications of the serial dilution method [37]. From this perspective, the report of Schneider et al. (2013) seems to be relevant for comparison due to the same method being utilized for the investigation of the antibacterial activity of Manuka and Portobello honeys. The researchers found that honeys diluted to 50% and 75% in tryptic soy broth significantly inhibited the growth of *E. coli*, *P. aeruginosa*, and *S. aureus*. Their study demonstrated that *E. coli* (NCTC 10418) was the most resistant to a 10% dilution of Manuka honey compared to other studied microbes [38]. Brudzynsky et al. (2006) used a broth microdilution assay for the analysis of the antimicrobial characteristics of a wide range of Canadian honeys against *E. coli* (ATCC 14948) and *Bacillus subtilis* (*B. subtilis*) (ATCC 6633). The authors reported that all tested samples of honey at a 64-fold dilution maintained antibacterial activity with higher selectivity against *E. coli*, whereas *B. subtilis* totally lost sensitivity to the solvents at this concentration. Also, it was concluded that antimicrobial properties might be associated with H_2_O_2_, the plant-specific composition of honey, and the possible presence of *E. coli*-specific compounds [17]. Lu et al. (2013) investigated the antibacterial characteristics of Manuka and Kanuka honeys, Manuka–Kanuka blends, and clover honey against *E. coli* O157:H7, *B. subtilis* 168, *S. aureus* ATCC 25923, and *P. aeruginosa* PAO1 (ATCC 15692) using a broth microdilution assay. In this study, *P. aeruginosa* revealed less sensitivity to all honey samples. Results were explained by the floral-specific phenolic structure of honey, bee-derived antimicrobial peptides, and the hypothetical synergistic effects of honey compounds [39]. 

Phloretin, at a 7 mg/mL concentration, did not significantly reduce the growth of *E. coli.* Furthermore, at 2 mg/mL concentration, phloretin was associated with the excessive growth of *E. coli* in all rounds of the experiment. A penicillin–streptomycin solvent was utilized as a positive control, exerting significant inhibitory action against *E. coli*. Regarding the results of phloretin testing in our study, it is important to define the potential role of honey and its extracts as a substrate for bacteria. The study conducted by Schneider et al. (2013) revealed that Manuka and Portobello honeys at 1% (*v*/*v*) solution induced the overgrowth of *S. aureus* [38]. There is a shortage of studies discussing these kinds of results and explaining the possible molecular mechanisms behind the stimulatory effects of honey on bacterial growth.

The antimicrobial activity of honey phytochemicals was also tested using two variances of diffusion assay. First, the antimicrobial effects against *E. coli* were determined for PLA, PCA, and phloretin at 100 μg per disc. None of the honey constituents had visible zones of inhibition. Then, the antibacterial capacity of honey compounds was investigated at 350 μg per disc. Again, all studied phytochemicals at increased concentration did not exhibit antibacterial action against *E. coli*. Further, the antimicrobial effects of the investigated phytochemicals at 700 μg per well were examined using a well diffusion assay. Similarly, none of the phytochemicals had an inhibitory effect against *E. coli*. According to Akujobi et al. (2010), the well diffusion assay showed more accuracy in the analysis of the sensitivity of *E. coli* and *S. aureus* to Nigerian honey in comparison with the disc diffusion assay [40]. 

In addition, the effects of penicillin–streptomycin in cooperation with PCA at 2 mg/mL concentration showed that this phytochemical demonstrates a significant decrease in the growth of *E. coli*, supporting the inhibitory action of the antibiotics. This finding supports the hypothesis that some honey constituents may increase the bactericidal or bacteriostatic effects of conventionally used antibiotics. It might be suggested that other honey phytochemicals could be tested for their antimicrobial-enhancing properties using this method.

The antibacterial properties of honey have been extensively documented in numerous research studies [32]. The components found within honey exhibit varying degrees of activity against a wide array of microorganisms, including pathogens that have developed resistance to conventional antibiotics [34]. The scanning electron microscope investigations undertaken by Dieuleveux et al. (1998) revealed that 7 mg/mL of PLA impedes the growth of *L. monocytogenes*, resulting in the loss of rigidity in their cell walls [41]. Studies involving Manuka honey have revealed its effectiveness against pathogens such as *S. aureus*, *Salmonella typhimurium*, *E. coli*, *P. aeruginosa*, and *B. subtilis* [5,39]. Furthermore, certain in vivo investigations have indicated that honey may also impede the growth and proliferation of methicillin-resistant *S. aureus* (MRSA) and vancomycin-resistant enterococci [42,43]. Despite MGO being identified as the primary antimicrobial component of Manuka honey, its antibacterial properties may also be elucidated by its phenolic compounds [44]. Thus, PLA demonstrates amphiphilic properties attributable to its chemical structure, featuring a hydrophobic benzene ring and a hydrophilic carboxy group. This configuration facilitates its interaction with lipid molecules and proteins within the cell membrane, leading to the disruption of membrane permeability and integrity. Moreover, fluorescence assays have indicated that PLA can interact with bacterial genomic DNA through intercalation, suggesting a dual antibacterial mechanism targeting both the membrane and genomic DNA [23]. PCA also demonstrates antimicrobial activity through multiple mechanisms. Initially, phenolic acids disrupt the structural integrity of the bacterial cell wall by altering its rigidity and modifying the dynamics of the phospholipid bilayer membrane. This process directly results in increased membrane permeability and the leakage of cytoplasmic compounds [45]. Moreover, PCA not only disrupts the barrier function of the cell wall but also affects the DNA of microorganisms [24]. Besides the described mechanism of bacterial wall damage, it has been observed that minimum inhibitory concentrations, ranging from 10 to 80 mg/mL, have the ability to bind to the phosphate anion in the DNA double helix and intercalate within the groove of the DNA double helix [27]. The antibacterial effect of phloretin remains incompletely investigated. Certain authors propose that this compound impedes biofilm formation and pathogenicity by targeting virulence factors. Furthermore, its influence on bacterial toxins like endotoxin/LPS, achieved through a mechanism that inhibits their interaction with cellular receptors such as TLRs, represents another mode of action for its anti-inflammatory effects linked to bacterial infection [46]. Table 4 delineates the principal discoveries derived from primary investigations concentrating on the antimicrobial attributes of specific phenolic compounds against *Escherichia coli*.

Our study possesses both strengths and limitations. The main limitation of our study is its focus on a single bacterial species, specifically *E. coli.* Nevertheless, our discoveries hold the potential to significantly advance the ongoing inquiry into novel antimicrobial agents targeting resistant bacterial strains. A notable strength of our study lies in its utilizing a broth culture assay to illustrate the antimicrobial properties of phytochemicals, alongside a comparison with an agar diffusion assay. Additionally, our experiments involving the combination of PCA with antibiotics are entirely novel and distinctive. 

## 4. Materials and Methods

### 4.1. Bacteria

The bacterial strain of *E. coli* ATCC 25922 grown on the agar plate with 5% sheep blood agar was from the Department of Microbiology Laboratory, Republican Diagnostic Center, Corporate Fund “University Medical Center”, Astana. The plates inoculated with the strains were placed in an incubator set at 35 ± 2 °C under aerobic conditions enriched with carbon dioxide. After an incubation period of 18 to 24 h, the plates containing the strains were assessed for growth quantity, colony dimensions, and hemolytic reactions [50]. 

### 4.2. Honey Constituents

3-phenyllactic acid (α-Hydroxyhydrocinnamic acid), *p*-coumaric acid (trans-4-Hydroxycinnamic acid), and phloretin (3-(4-hydroxyphenyl)-1-(2,4,6-trihydroxyphenyl)-1-propanone) were purchased from Sigma-Aldrich (St. Louis, MO, USA). All components exhibit a purity of ≥98.0%.

### 4.3. Comparison of the Antimicrobial Activity of Honey Constituents In Vitro Using Broth Culture Assay

A broth culture assay was utilized to identify the inhibitory action of honey constituents against *E. coli* ATCC 25922. The method of Schneider et al. (2013) was used with some modifications [38]. A solution was prepared by combining 5 mL of tryptic soy broth (TSB) with 1 mL of methanol/water solvent (20%/80%) containing either 2 mg/mL of 3-phenyllactic acid, or 2 mg/mL of *p*-coumaric acid, or 2 mg/mL of phloretin. Negative controls included the use of 5 mL TSB alone and 5 mL TSB mixed with 1 mL of methanol/sterile water solvent (20%/80%). Also, TSB with penicillin–streptomycin (P0781, Sigma-Aldrich, USA) was prepared as a positive control (2000U penicillin; 2 mg/mL streptomycin). All samples were inoculated with 100 μL of an overnight starting culture of *E. coli*. Inoculated broths were incubated aerobically for 24 h at 37 °C with shaking in an orbital shaker. Broths were sampled and serially diluted using 0.1 M phosphate-buffered saline (PBS). A sample of 1 mL was added to 9 mL of PBS and then serially diluted to obtain dilutions such as ×10, ×100, ×1000, ×10,000, ×100,000, ×1,000,000, and so forth. Each sample was taken as 100 μL and spread onto TSA plates. Plates that had between 30 and 300 cfu were counted. All readings were made in duplicate, and experiments were conducted on 3 separate occasions. The same procedures were performed for testing 7 mg/mL of 3-phenyllactic acid, 7 mg/mL of *p*-coumaric acid, and 7 mg/mL of phloretin.

### 4.4. Measurement of the Inhibitory Properties of Honey Constituents In Vitro Using Diffusion Assays

#### 4.4.1. Disc Diffusion Assay

The method of Kirkpatrick et al. (2017) was adapted with some modifications [47]. A total of 100 µL of an overnight liquid culture of *E. coli* (at a turbidity of 0.5 McFarland standard) was spread evenly using a sterile spreader and allowed to dry. Three sterile blank discs (74146-25DISCS-F, Sigma-Aldrich, USA) were placed on each nutrient agar plate using sterile forceps. Briefly, 100 μg of each phytochemical diluted in methanol/sterile water solvent (20%/80%) was prepared per disc. As a negative control, 50 μL of methanol/sterile water solvent (20%/80%) was tested, whereas the 100U penicillin/100 μg streptomycin and 400 μg of MGO per disc were used as positive controls. Nutrient agar plates were incubated at 37 °C for 24 h. The diameter of the zones of inhibition was measured using a clear ruler. Tests were performed in duplicate, and experiments were repeated on 3 separate occasions. The same procedures were performed for testing phytochemicals at a concentration of 350 μg per disc. 

#### 4.4.2. Well Diffusion Assay

A 24 h liquid culture of *E. coli* was spread evenly using a sterile spreader on the nutrient agar plates and allowed to dry. The turbidity of bacterial suspension was adjusted using 0.5 McFarland standard. Three wells per nutrient agar plate were placed using a cork borer. A total of 100 µL of each phytochemical diluted in methanol/sterile water solvent (20%/80%) (700 μg of each phytochemical per well) was placed in the wells. The assay also involves methanol/sterile water solvent (20%/80%) as a negative control. The methanol/sterile water solvents (20%/80%) of 200U penicillin/200 μg streptomycin and MGO (800 μg) were used as positive controls. The nutrient agar plates were incubated at 37 °C for 24 h, and the diameter of the zones of inhibition was measured. Results are an average of triplicate observations. Experiments were repeated on 3 separate occasions.

### 4.5. Investigation of the Effects of Sub-Inhibitory Concentrations of p-Coumaric Acid on the Susceptibility of E. coli ATCC 25922 to Penicillin-Streptomycin

Additionally, the susceptibility of *E. coli* incubated with a subinhibitory (2 mg/mL) concentration of *p*-coumaric acid to penicillin–streptomycin was checked. A total of 2 mg/mL *p*-coumaric acid was made in 4 mL of TSB. A total of 5 mL of TSB only and 4 mL of TSB containing the methanol/sterile water solvent (20%/80%) were used as negative controls. Each tube was inoculated with the overnight starting culture of *E. coli* and incubated for 24 h at 37 °C in the shaker at 120 rpm. The nutrient agar plates were spread evenly over the surface with 100 µL of samples from the broths. The sterile paper disks were placed at equal distances onto the plates. Each sterile disk was loaded with 50 µL of 100U penicillin/100 µg streptomycin. The plates were inverted and placed in the incubator for 24 h at 37 °C. Zones of inhibition were measured with a clear ruler.

### 4.6. Statistical Analysis

All experiments were conducted in duplicate on three/four separate occasions by the principles of statistical analysis and experimental reproducibility. Data were presented as the mean with the standard error (SEM) or standard deviation (SD). To test the significance, a two-tailed independent Student’s t-test was used. A *p*-value of ≤0.05 was considered statistically significant. The analysis was performed in Microsoft Excel 2010.

## 5. Conclusions

Using a broth culture assay, the antibacterial properties of 3-phenyllactic acid, *p*-coumaric acid, and phloretin—phytochemicals previously identified in honey—against *E. coli* (ATCC 25922) were demonstrated. PLA was the most efficient antimicrobial agent, followed by PCA, whereas phloretin, at lower concentrations (2 mg/mL), led to the excessive growth of *E. coli*. Despite expectations, various modifications of agar diffusion assays did not reveal the antibacterial properties of the studied phytochemicals. Furthermore, a sub-inhibitory concentration of PCA enhanced the antimicrobial activity of penicillin–streptomycin. These findings might be helpful for the further investigation and development of new antimicrobial agents for the treatment of skin infections and wounds.

## Figures and Tables

**Table 1:** Antimicrobial activity of honey phytochemicals at 100 μg per disc measured using an agar disk diffusion assay. Results are expressed as mean zone of inhibition (mm) ± standard deviation (SD).

Phytochemical/Control	Mean Zone of Inhibition (mm) ± SD
3-Phenyllactic Acid (100 µg per disk)	0.0 ± 0.0
*p*-Coumaric Acid (100 µg per disk)	0.0 ± 0.0
Phloretin (100 µg per disk)	0.0 ± 0.0
Pen/Strep (100U/100 µg per disc)	22.0 ± 0.0
Methylglyoxal (400 µg per disc)	26.0 ± 0.0
Negative Control (Methanol–Water, 20:80)	0.0 ± 0.0

**Table 2:** Antimicrobial activity of honey phytochemicals measured at 350 μg per disc using an agar disk diffusion assay. Results are expressed as mean zone of inhibition (mm) ± standard deviation (SD).

Phytochemical/Control	Mean Zone of Inhibition (mm) ± SD
3-Phenyllactic Acid (350 µg per disk)	0.0 ± 0.0
*p*-Coumaric Acid (350 µg per disk)	0.0 ± 0.0
Phloretin (350 µg per disk)	0.0 ± 0.0
Pen/Strep (100U/100 µg per disc)	28.0 ± 0.0
Methylglyoxal (400 µg per disc)	22.0 ± 0.0
Negative Control (Methanol–Water, 20:80)	0.0 ± 0.0

**Table 3:** Antimicrobial activity of honey phytochemicals measured using an agar well diffusion assay. Results are expressed as mean zone of inhibition (mm) ± standard deviation (SD).

Phytochemical/Control	Mean Zone of Inhibition (mm) ± SD
3-Phenyllactic Acid (700 µg per well)	0.0 ± 0.0
*p*-Coumaric Acid (700 µg per well)	0.0 ± 0.0
Phloretin (700 µg per well)	0.0 ± 0.0
Pen/Strep (200U/200 µg per well)	33.5 ± 2.2
Methylglyoxal (800 µg per well)	31.8 ± 1.7
Negative Control (Methanol–Water, 20:80)	0.0 ± 0.0

**Table 4:** Key studies regarding the antimicrobial effects of some phenolic compounds against *E. coli*.

Author	Year	Main Findings	References
Schneider et al.	2013	This study compared locally produced Portobello honey (PBH) with Manuka honey (MH) in fighting three bacteria causing wound infections. Both honeys demonstrated significant inhibition at 75% and 50%, although PBH at 10% had slightly lower activity than MH (*p* ≤ 0.001).	[38]
Kirkpatrick et al.	2017	This study compared the antibacterial, phenol content, and antioxidant abilities of MSY, MGO, and PLA from Manuka honey. Antioxidant capacity was tested with ABTS or IRAC, while antibacterial effects were measured against *E. coli*, *B. subtilis*, or *S. aureus* using a disc diffusion assay. MGO and PLA showed antibacterial effects but lacked noticeable antioxidant or phenol traits.	[47]
Lou et al.	2011	This study evaluated PCA’s antibacterial activity and mechanism against bacteria, finding effective inhibition of bacterial growth at MIC values from 10 to 80 mg/mL. The results suggest that PCA acts by disrupting cell membranes and binding to bacterial DNA, leading to cell death.	[27]
Elamine et al.	2021	Zantaz honey’s antibacterial effects on *E. coli*, *P. aeruginosa*, and *S. aureus* were assessed using chemometric tools. Polyphenols, particularly epicatechin, 4-coumaric acid, methylsyringate, and PCA, showed a strong positive correlation with antibacterial activity.	[48]
Kot et al.	2015	This study evaluated phytochemicals as potential alternative antimicrobials for preventing and deactivating *E. coli* biofilms on urinary catheters. Phytochemicals could serve as significant sources of antibiofilm agents with preventive capabilities against *E. coli* biofilm formation on urinary catheters.	[49]
Ning et al.	2017	This study investigated PLA’s antibacterial mechanism against *L. monocytogenes* and *E. coli.* Flow cytometry using propidium iodide (PI) showed PLA’s ability to damage *L. monocytogenes’* membrane but not *E. coli’s*. A fluorescence assays indicated PLA’s interaction with bacterial DNA through intercalation, suggesting that it targets both membrane and genomic DNA.	[23]

## Data Availability

The datasets used and/or analyzed during the current study are available from the corresponding author on reasonable request.
